# Suicidal selection: Programmed cell death can evolve in unicellular organisms due solely to kin selection

**DOI:** 10.1002/ece3.5460

**Published:** 2019-07-30

**Authors:** Anya E. Vostinar, Heather J. Goldsby, Charles Ofria

**Affiliations:** ^1^ Grinnell College Grinnell Iowa; ^2^ Michigan State University East Lansing Michigan

**Keywords:** artificial life, kin selection, programmed cell death

## Abstract

**Abstract:**

Unicellular organisms can engage in a process by which a cell purposefully destroys itself, termed programmed cell death (PCD). While it is clear that the death of specific cells within a *multicellular* organism could increase inclusive fitness (e.g., during development), the origin of PCD in *unicellular* organisms is less obvious. Kin selection has been shown to help maintain instances of PCD in existing populations of unicellular organisms; however, competing hypotheses exist about whether additional factors are necessary to explain its origin. Those factors could include an environmental shift that causes latent PCD to be expressed, PCD hitchhiking on a large beneficial mutation, and PCD being simply a common pathology. Here, we present results using an artificial life model to demonstrate that kin selection can, in fact, be sufficient to give rise to PCD in unicellular organisms. Furthermore, when benefits to kin are direct—that is, resources provided to nearby kin—PCD is more beneficial than when benefits are indirect—that is, nonkin are injured, thus increasing the relative amount of resources for kin. Finally, when considering how strict organisms are in determining kin or nonkin (in terms of mutations), direct benefits are viable in a narrower range than indirect benefits.

**Open Research Badges:**

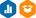
This article has been awarded Open Data and Open Materials Badges. All materials and data are publicly accessible via the Open Science Framework at https://github.com/anyaevostinar/SuicidalAltruismDissertation/tree/master/LongTerm.

## 1 INTRODUCTION

In programmed cell death, a cell destroys itself through internally controlled processes (Nedelcu, Driscoll, Durand, Herron, & Rashidi, 2011). Initially, it may seem puzzling that such apparently undesirable behavior has not been eradicated by natural selection (Nedelcu et al., 2011). However, if the cell is part of a multicellular organism, programmed cell death has the potential to increase the survival of the overall organism. As long as the remaining cells that benefit are kin to the destroyed cell (as is the case for most multicellular organisms), this behavior will be under positive selection (Jacobson, Weil, & Raff, 1997). For example, the purging of damaged or diseased cells to maintain health, or the restructuring of tissue to facilitate normal development (Kerr, Wyllie, & Currie, 1972). However, many types of behavior resembling programmed cell death have also been identified in unicellular organisms (Koonin & Aravind, 2002). This situation is less clear given that programmed cell death in a unicellular organism kills the entire organism, eliminating any direct selective pressure for this trait. This observation raises the question: How can programmed cell death evolve at all in unicellular organisms?

One adaptive explanation for programmed cell death in unicellular organisms is that within a spatially structured environment, the co‐located surviving kin receive some benefit from the death (Foster, Wenseleers, & Ratnieks, 2006). However, alternative nonadaptive hypotheses for the origin of programmed cell death have recently been proposed (Durand, Sym, & Michod, 2016; Nedelcu et al., 2011). These alternative hypotheses include: (a) programmed cell death is a side effect of behavior evolved in previous environments, (b) programmed cell death is caused by a hitchhiking and/or pleiotropic gene wherein a beneficial trait offsets this undesirable behavior, or (c) programmed cell death is a pathological breakdown of the cell's functioning and is not under selection due to benefits provided to kin. Finally, there is the historically accepted scenario of kin selection [where selection for programmed cell death is due to the benefits provided to kin instead of directly benefiting the organism itself (Foster et al., 2006)].

Determining the likelihood that these scenarios resulted in the evolution of programmed cell death is difficult for a number of reasons: Scenarios one and two require knowledge of past evolutionary conditions to evaluate definitively; unfortunately, this detailed knowledge does not exist for many ancient organisms. Testing scenario three would require experimental evolution treatments that control whether a benefit is provided to kin or not. This is difficult to accomplish in an organic system due to both time constraints and current technological capabilities.

Because there is no simple way of evaluating these alternative hypotheses using historical data or within organic systems, we use self‐replicating digital organisms, a type of computational modeling that evolves agents in a complex digital environment, to determine whether programmed cell death could evolve due to kin selection without any other possible scenarios. For this study, we use Avida, which is a digital evolution platform used to study evolutionary questions, including complexity (Lenski, Ofria, Pennock, & Adami, 2003), division of labor (Goldsby, Dornhaus, Kerr, & Ofria, 2012), and communication (Beckmann & McKinley, 2009). An Avida experiment consists of a virtual world where digital unicellular organisms, or “unicells,” can evolve for thousands of generations over hours with perfect control and data collection. Using Avida, we implemented programmed cell death behavior. We then observed under what conditions programmed cell death was able to evolve into a population de novo.

Our hypothesis is that programmed cell death can evolve as a response to specific types of kin selection. For this work, we categorize the potential benefits of programmed cell death into two major categories: direct benefit to kin and indirect benefit to kin through harm to competitors. Indirectly beneficial programmed cell death, when an organism dies, its surrounding kin are provided a competitive advantage. For example, in *Dictyostelium discoideum* individual organisms die in order to form a stalk that lifts the spores of those remaining for better propagation (Strassmann & Queller, 2011). In indirectly beneficial programmed cell death, when an organism dies the surrounding nonkin are set at a competitive disadvantage, such as colicin production in *Escherichia coli*, which kills any organisms without the resistance gene after the focal organism has burst (Chao & Levin, 1981). These two forms of programmed cell death occur throughout nature, but are difficult to directly compare due to the need to control for the many other differences between species that exhibit one form or the other (Bidle, 2016; Durand et al., 2016; Smith, 1980). While there has been ample work using agent‐based models to study the evolution of altruism (Gotts, Polhill, & Law, 2003), there are fewer studies using this method to explore programmed cell death specifically. Libby et al. explored the benefits of PCD for unicellular organisms using a specific type of indirect benefit and found that PCD can persist in a population under those conditions (Libby, Driscoll, & Ratcliff, 2018). Previously, we have examined the effect of population size and mutation rate on the most extreme form of indirect benefit: killing competing organisms (Johnson, Goldsby, Goings, & Ofria, 2014). Here, we analyze the potential benefits of PCD using more general benefits and do not consider differing population sizes or mutation rates.

Using the Avida software, we created two forms of programmed cell death that differed only in whether the effect directly increased the fitness of kin or decreased the fitness of competitors. We provide experimental evidence that either type of benefit to kin can be sufficient to cause the evolution of programmed cell death. Furthermore, we found that direct benefits led to a larger proportion of the population with the programmed cell death behavior in some replicates. However, direct benefits also led to some replicates not evolving programmed cell death behavior at all in conditions where the indirect benefits always led to some use of programmed cell death. Finally, we found that indirect benefits led to a different pattern of response to the degree of kin discrimination than direct benefits. These findings are critical to our understanding of the evolution of programmed cell death as the form of benefit directly changes the conditions under which it will evolve.

## 2 AVIDA DIGITAL EVOLUTION SYSTEM

**Figure 1:**
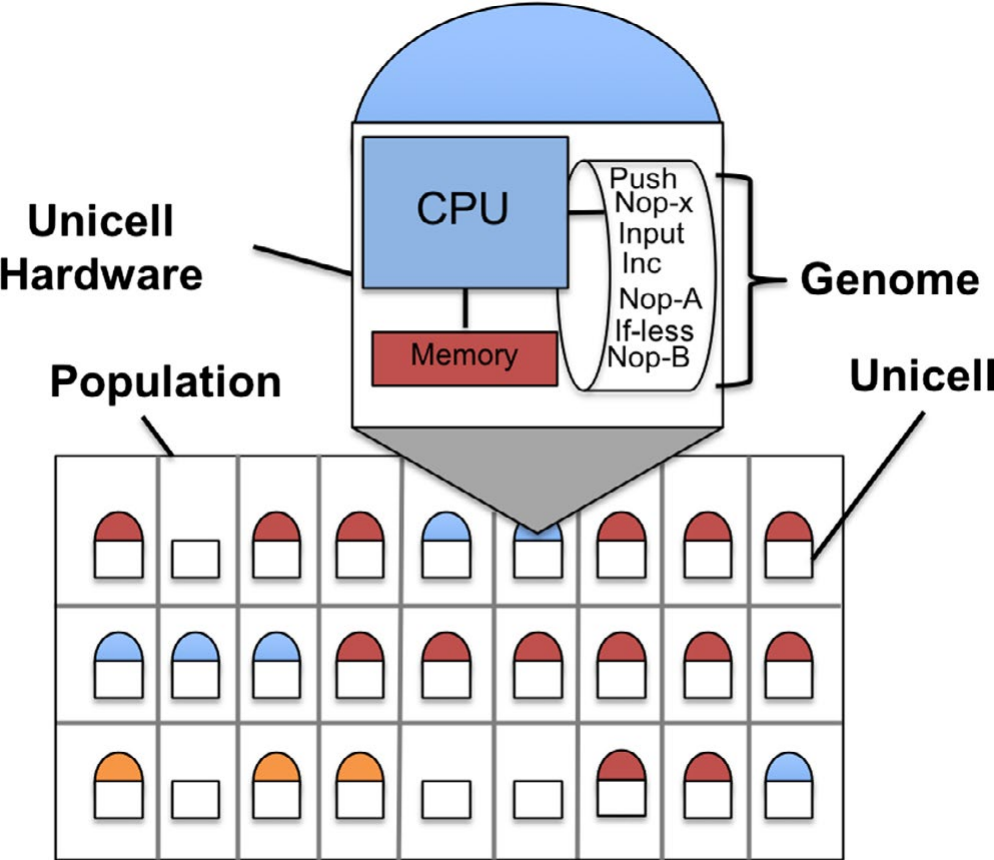
A simplified example of an Avida world. Half‐circles are unicells where color indicates differences in genomes. One unicell's internal hardware is shown, including CPU, memory, and a genome of program instructions

For this study, we use the Avida digital evolution system (Ofria, Bryson, & Wilke, 2009). Avida is a flexible system that has been used for evolutionary studies with different biological analogies, including bacterial cells (Beckmann & McKinley, 2009) and eusocial insects (Goldsby et al., 2012). Here, we consider each digital organism to be the digital equivalent of a unicellular organism, which we call “unicells.” As shown in Figure 1, Avida consists of a virtual world where unicells compete for space as they reproduce with variation. Each grid position may contain a single unicell, which can replicate into the eight neighboring grid positions.

Each unicell has a genome consisting of computational instructions from a Turing‐complete programming language (that is it can theoretically process any computable function), a portion of a genome is shown in Figure 1. Each unicell also has a virtual CPU on which its genome is executed. This CPU includes storage space, which consists of registers and stacks.

A unicell is able to reproduce by executing a series of instructions that fully copies its genome, followed by a “divide” instruction to place the resulting offspring into one of eight immediately neighboring spaces, killing any organism that was occupying that space. This copying process is imperfect, however, and mutations may be introduced. The rate at which these mutations occur is based on user‐configured settings. These mutations produce the required inheritable variation for selection to act on, and when combined with competition for limited space, lead to evolution by natural selection. Within Avida, the metabolic rate of a unicell determines how many CPU cycles it is given compared to the rest of the population. As such, metabolic rate is similar to the amount of energy a natural organism has to forage. A unicell may receive a bonus through the programmed cell death of another unicell. This bonus increases the metabolic rate (and thus number of CPU cycles) of any offspring the unicell has. These extra cycles will allow the offspring to execute its genome faster, and therefore potentially reproduce faster, allowing its genotype to spread more quickly through the population. Conversely, a unicell that is harmed by the programmed cell death of a nonkin unicell will have offspring with a lower metabolic rate (receiving fewer CPU cycles), which will therefore reproduce more slowly. Unicells cannot die of old age, they can only be killed by being overwritten when another unicell reproduces. We used the default settings of Avida [found in Ofria & Wilke (2004)] with a few changes. First, we added two novel programmed cell death behaviors described below. Second, we disabled the default logic tasks that a unicell could be rewarded for completing, meaning that the unicells cannot increase their metabolic rate in anyway other than by being gifted it from a related organism that executed programmed cell death. Third, we removed the possibility of insertion or deletion mutations so that organisms remain at exactly 100 instructions in length.

### 2.1 Programmed cell death instructions

We implemented two programmed cell death instructions to test the importance of direct versus indirect kin selection: The *direct‐kin‐benefit programmed cell death instruction (direct‐pcd)*, when executed successfully, kills the unicell, but increases the metabolic rate of a unicell's kin that are nearby. In contrast, the *indirect‐kin‐benefit programmed cell death instruction (indirect‐pcd)*, when executed successfully, decreases the metabolic rate of any unrelated (nonkin) unicells that are nearby. To enable us to compare the effects of direct versus indirect kin benefits, we made the behavior of both types of instructions either directly increase the metabolic rate of kin or directly decrease the metabolic rate of nonkin. These two forms of programmed cell death are high‐level abstractions of the many ways programmed cell death can affect surrounding organisms in organic systems, such as producing a toxin, producing an external enzyme necessary for survival, or removing the threat of a bacteriophage from the colony (Refardt, Bergmiller, & Kummerli, 2013).

When a unicell attempts to execute one of the programmed cell death instructions (either direct‐pcd or indirect‐pcd), there is a 5% chance the instruction is successful and that the unicell will kill itself and cause benefit or harm to surrounding unicells in a 2‐space radius on the grid. Clearly, the effects of this behavior hinge upon which surrounding unicells are considered kin and which are not. There are two aspects to this determination of kin: “kin inclusivity level” (i.e., how many genetic differences are tolerated in kin) and kin recognition (i.e., how accurate a unicell is at identifying related organisms).

**Figure 2:**
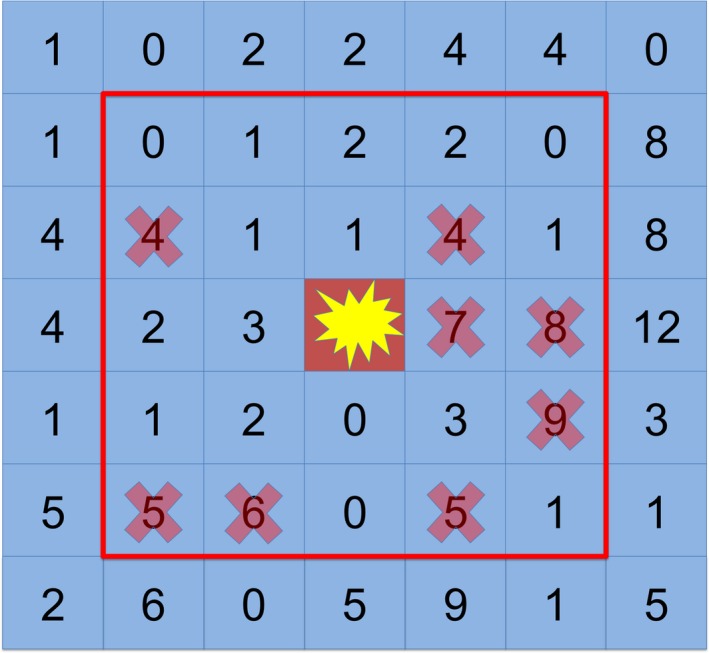
An example of a unicell undergoing programmed cell death. Each square represents a unicell. The number in each cell is a number of genetic differences between the unicell in that space and the focal unicell depicted as an explosion. Every unicell is a 2‐space radius (red line) is evaluated as kin or nonkin. Nonkin are marked with red X's. If the programmed cell death has direct benefit, unicells within the radius without a red X will receive the benefit. If the behavior has indirect benefit, unicells with red X's will be harmed

We define “kin inclusivity level” (KIL) to be a measure of how distantly related organisms may be before they are considered nonkin by the programmed cell death instructions. Specifically we use the number of genetic differences between two unicells to measure kinship. For example, a kin inclusivity level of three – the default in our experiments – means that a nearby unicell whose genome has up to three genetic differences from the unicell executing the programmed cell death instruction will be considered kin (see Figure 2). In experiments with direct benefits (direct‐pcd‐3), this unicell's kin's metabolic rate will be multiplied by 5. Conversely, in experiments with indirect benefits (indirect‐pcd‐3), any unicell in the radius with more than three genetic differences will have its metabolic rate divided by 5. The ancestor unicell's metabolic rate is initially the size of its genome, in this case 100. Therefore, unicells with a benefit will on average be able to copy their entire genome and produce an offspring before unicells without the benefit are able to copy one fifth of their genome. Conversely, unicells with the penalty will, on average, only be able to execute instructions at a rate of one fifth compared to nonpenalized unicells. This effect is extreme with the goal of reflecting systems in which the focal organism releases colicins to kill nearby competitors or destroys a virus that would otherwise be deadly to surrounding kin (Refardt et al., 2013).

Within our system, kin recognition by unicells is automated as part of the execution of the programmed cell death instructions. In particular, kin recognition within this system is perfect and uses the number of genetic differences between two unicells to compute kinship. The kin distance is the Hamming distance between the focal unicell and every other unicell in a 2‐cell radius. Hamming distance is the measure of the number of differences between genomes when genomes are a fixed size. To decrease computational overhead, we required all unicells to have a genome of length 100, allowing for Hamming distance to be used instead of Levenshtein distance. This kin‐recognition system is an idealized form of the many kin‐recognition systems found in organic organisms (Ho, Hirose, Kuspa, & Shaulsky, 2013) and can be thought of as focusing on, for example, only the genes that encode a particular external protein or the presence of a plasmid (Chao & Levin, 1981).

## 3 METHODS

Here, we outline the factors we considered in the de novo evolution of both forms of programmed cell death. We ran each experiment for 60,000 updates^1^ or approximately 2,000 generations. For every treatment, we ran 30 replicates. Avida is open‐source at: https://github.com/devosoft/avida and our implementation is at commit 102dd2071558ccd981a2cdab7af47f99ff8d4299. All analysis scripts and data are at https://github.com/anyaevostinar/SuicidalAltruismDissertation/tree/master/LongTerm.

### 3.1 Kin inclusivity level

Our previous work has suggested that the level of kin discrimination can greatly alter the benefits of programmed cell death (Johnson et al., 2014). If too many unicells are considered kin (i.e., kin inclusivity level is high and unicells with many genetic differences are considered kin), cheating unicells that do not possess the programmed cell death gene may gain the benefit, decreasing the relative inclusive fitness for that gene. [Inclusive fitness is a measure of fitness that takes into account all copies of that unicell's genetic variants in the population, including its siblings and their offspring, e.g., Taylor (1992)]. However, if too few unicells are considered kin (i.e., kin inclusivity is low and few genetic differences are tolerated), mutants that contain the programmed cell death gene or contain other beneficial traits are considered nonkin and thus fail to invade, decreasing the population's evolvability.

To explore how kin inclusivity levels influence the evolution of programmed cell death, we tested a wide range of kin inclusivity levels (KIL): 0, 1, 3, 5, 30, and 100. Because our unicells were required to have genomes of length 100, these treatments span the full possible range of kin inclusivity from only clones being considered kin (KIL 0) to every possible unicell being considered kin (KIL 100).

### 3.2 No‐effect controls

To assess whether kin selection can be the sole driver of the evolution of programmed cell death, we ran controls for every replicate where the programmed cell death killed the unicell but had no other direct or indirect effect on kin. In the experimental treatments, the effect is 5: for direct benefits, metabolic rate of kin was multiplied by 5; for indirect benefits, metabolic rate of nonkin was divided by 5. In our controls, we set the effect to the identity 1, so that nothing changed in our configurations except the effect to metabolic rate. This control allows us to measure the exact effect of there being a benefit – direct or indirect – to kin on the evolution of programmed cell death.

## 4 RESULTS AND DISCUSSION

**Figure 3:**
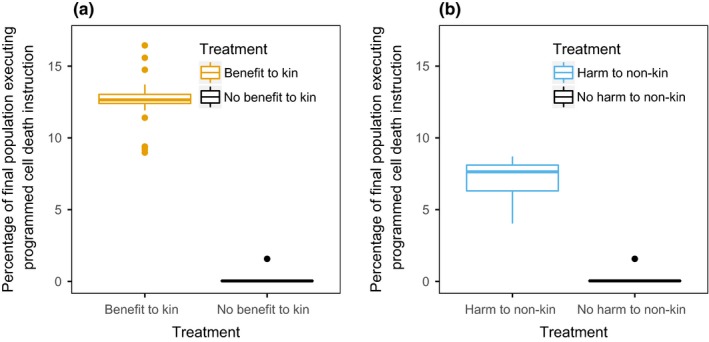
(a) When there is a direct benefit to kin, 12.52% of the population attempt to perform the programmed cell death behavior. However, when that benefit is removed, the behavior does not evolve into the population. (b) When there is an indirect benefit to kin, 7.11% of the population attempt to perform the programmed cell death behavior. However, when the indirect benefit is removed, the behavior does not evolve into the population

The key question addressed by this study is as follows: Can programmed cell death evolve strictly due to the benefit conferred to surrounding kin? To test this question, we created an environment where it was not possible for unicells to die due to pathology, gain fitness through other tasks, or maintain adaptations to a previously different environment. We then made a programmed cell death instruction (direct‐pcd) available via mutation. This programmed cell death behavior was stochastic in that it had only a 5% probability of successfully killing the unicell and conferring benefits. Successful use of this behavior increased resources of surrounding kin unicells. We compared the amount that the behavior was attempted when given this benefit to kin to how much it was attempted when no benefit was given to kin (a control). As shown in Figure 3a, we found that 12.52% of unicells executed the programmed cell death instruction when there was a benefit to surrounding kin, compared to 0.09% when there was not a benefit to surrounding kin (pairwise Wilcox test *p* < 2.2e‐16). This result demonstrates that under these environmental conditions, kin selection is sufficient as the sole selective pressure for the evolution of programmed cell death.

### 4.1 How do indirect benefits affect programmed cell death?

There is another category of programmed cell death, however, that could exhibit different evolutionary dynamics: programmed cell death that damages nonkin instead of directly benefiting kin. Many forms of programmed cell death involve the focal organisms producing harmful substances that only impact nonkin, such as colicinogenic *E. coli* (Chao & Levin, 1981), which was previously tested in Avida (Goings, Clune, Ofria, & Pennock, 2004). However, clearly such an indirect benefit only aids kin if nonkin are present within the environment. The indirect nature of this benefit could decrease the viability of kin selection for programmed cell death. To investigate this possibility, we created a second behavior that directly decreased the health of nonkin in the surrounding area, instead of directly increasing the resources of kin (indirect‐pcd). We again compared to a control treatment where there was no effect on surrounding nonkin or kin unicells. As shown in Figure 3b, we found that when there was an indirect benefit to kin resulting from harm to nonkin, 7.11% of unicells executed the programmed cell death instruction compared to 0.09% when there was no harm done to nonkin (pairwise Wilcox test *p* < 2.2e‐16). This result shows that an indirect benefit to kin is also sufficient for programmed cell death to evolve due to kin selection in conditions where it otherwise would not, in agreement with previous studies that suggested kin selection as a possible mechanism for this behavior (Ackermann et al., 2008; Fiegna & Velicer, 2005; Ostrowski, Katoh, Shaulsky, Queller, & Strassmann, 2008).

There are, however, differences in the evolution of programmed cell death when it provides direct benefits, as compared to when it provides indirect benefits. As seen when comparing Figure 3a,b, an indirect benefit reduces how beneficial programmed cell death is. When the benefit to kin is direct, 12.52% of unicells executed the programmed cell death instruction by the end of the experiment compared to 7.11% when the benefit was indirect (pairwise Wilcox test *p*‐value = 2.493e‐13). This nearly twofold difference in use demonstrates that the form of benefit significantly impacts the selection for programmed cell death and should be considered when analyzing organic systems. This result suggests that kin selection could favor a direct benefit, such as removing a fatal pathogen from the colony, a behavior found in one form of *E. coli* (Berngruber, Lion, & Gandon, 2013), over an indirect benefit as found in colicinogenic *E. coli* (Chao & Levin, 1981).

### 4.2 How does kin inclusivity level affect programmed cell death?

Finally, the accuracy and degree of kin discrimination determines how many unicells are considered kin or nonkin and therefore is likely to have a large impact on the evolution of programmed cell death under kin selection. To explore this behavioral factor, we varied the kin inclusivity level (the number of genetic differences necessary to qualify as nonkin) used for both direct and indirect benefits. As discussed in the Methods, our system uses perfect genetic information of length‐100 genomes to determine kinship and should be considered a simulation of a kin‐recognition system based on, for example, the form of an external protein (Chao & Levin, 1981).

**Figure 4:**
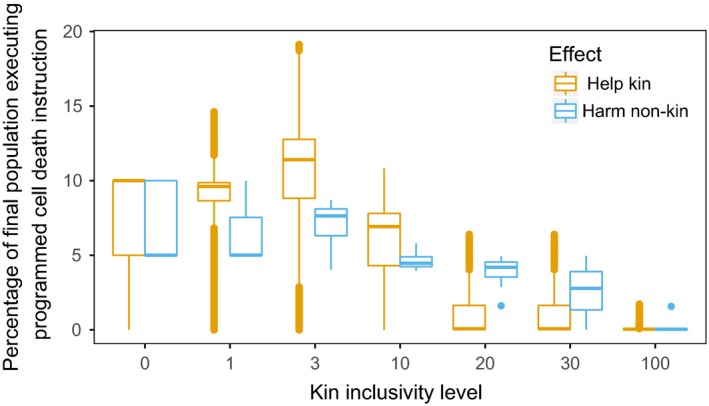
Percentage of population which performed programmed cell death when benefits to kin were direct or indirect with varying kin inclusivity levels. At direct‐pcd‐3, the programmed cell death behavior is used most frequently. For indirect benefits, a KIL 3 or lower leads to the most use of the behavior

As shown in Figure 4, we found that the kin inclusivity level has a significant effect on the evolution of programmed cell death for both direct and indirect benefits. When the benefit is direct, at a kin inclusivity level of zero, 7.58% of unicells execute the programmed cell death instruction on average compared to 0.05% when the kin inclusivity level is 100 (pairwise Wilcox test *p*‐value < 2.2e‐16).

Similarly, at a kin inclusivity level of zero, 7.33% of unicells execute the programmed cell death instruction with indirect benefits, compared to 0.09% when the kin inclusivity level was 100 (pairwise Wilcox test *p*‐value = 2.897e‐11). Note that the value of performing programmed cell death at kin inclusivity level zero is very low, as a result of a lack of variation in the population. At that level, mutants of any kind are severely penalized and therefore little genetic variation is able to persist. This result shows that programmed cell death can evolve due only to kin selection if there is an accurate and discriminatory form of kin recognition, though the exact degree of discrimination for an optimal benefit from programmed cell death clearly will rely on factors such as average mutation rate, as we have found previously (Johnson et al., 2014).

At intermediate kin inclusivity levels, the effect of kin selection on programmed cell death differs depending on whether benefits are direct or indirect (as shown in Figure 4). direct‐pcd‐3 was executed significantly more (10.32% of the unicells) than direct‐pcd‐0 (7.58% of unicells, pairwise Wilcox test *p*‐value < 2.2e‐16). However, the number of times the indirect‐pcd instruction was executed was not significantly different at indirect‐pcd‐3 (7.11% unicells) compared to indirect‐pcd‐0 (7.33% unicells, pairwise Wilcox test *p*‐value = 0.4639). Only at indirect‐pcd‐10 or higher is the instruction executed significantly less (4.67% unicells at indirect‐pcd‐10, pairwise Wilcox test *p*‐value = 6.637e‐09 compared to indirect‐pcd‐3). This result shows that the degree of kin discrimination differentially affects the likelihood of programmed cell death evolving via kin selection depending on whether the benefits to kin are direct or indirect.

### 4.3 What causes the different response to kin inclusivity level?

This difference between the use of programmed cell death in contexts where it provides direct and indirect benefits is due to the likelihood of the instructions having an effect on the neighboring unicells.

**Figure 5:**
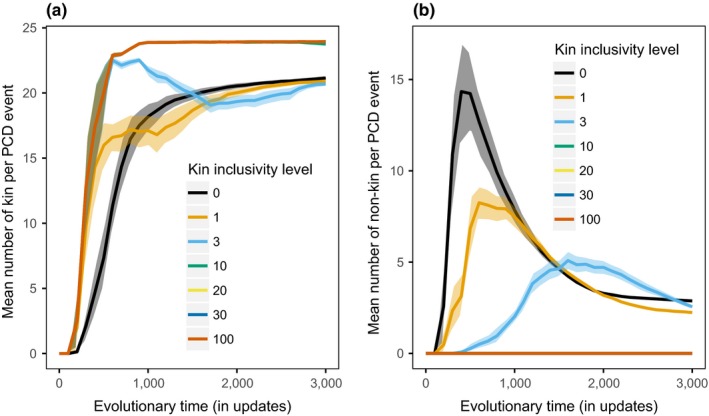
The average number of surrounding unicells directly affected per programmed cell death event across varying kin inclusivity levels when trait first emerges. (a) When benefits are direct, direct‐pcd‐100 have the highest amount of surrounding kin during initial evolution of the trait, leading to the most initial benefit. (b) When benefits are indirect, nonkin unicells are directly affected and at indirect‐pcd‐0, the most surrounding unicells are considered nonkin and therefore each programmed cell death event has the largest effect

**Figure 6:**
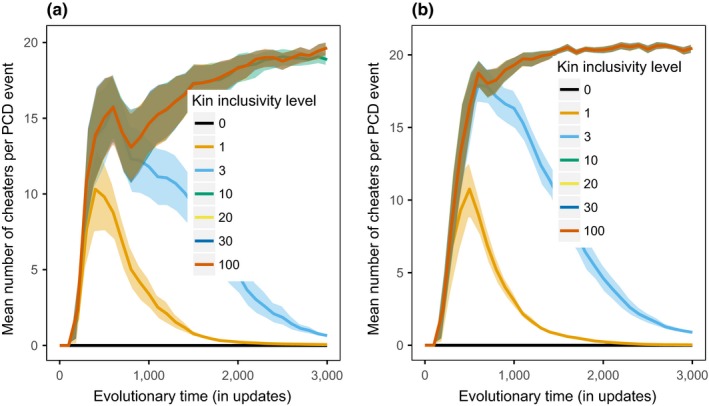
Number of cheating unicells, that is, unicells that are considered kin but do not contain the PCD instruction and therefore will never engage in the behavior. (a) While populations with direct‐pcd‐30 to 100 have a high initial benefit (Figure 5a), unicells without the PCD gene are considered kin throughout the experiment, decreasing the relative benefit of the PCD trait for unicells with the PCD gene. direct‐pcd‐3, however, enables unicells expressing the PCD trait to balance a high initial benefit with excluding unicells without the PCD gene after the initial emergence of the trait (cheaters). (b) At indirect‐pcd‐0, unicells without the PCD trait (cheaters) are prevented from invading completely throughout evolution. Unicells have no way of detecting presence of the PCD gene, the KIL requires unicells to use overall genetic difference as a proxy for the likelihood of another unicell having the PCD gene or not

When the benefit is direct, there is a trade‐off between the effectiveness of programmed cell death events during the initial invasion and the effectiveness of the programmed cell death events to resist subsequent invasion of unicell cheaters without the PCD gene. Initially, programmed cell death is most beneficial when a sufficient number of unicells are considered kin, as shown in Figure 5a. At direct‐pcd‐3, enough unicells are considered kin for a large benefit to be generated upon an individual's programmed cell death. If too few unicells are considered kin (such as at direct‐pcd‐0), there is significantly less average benefit generated per PCD event compared to direct‐pcd‐3 (at update 500 direct‐pcd‐0 mean kin unicells is 7.32, direct‐pcd‐3 kin unicells is 19.67, pairwise Wilcox test *p*‐value = 2.847e‐07). Later in evolution, if too many unicells are considered kin (such as at direct‐pcd‐100), unicells without the programmed cell death gene can gain a benefit from programmed cell death events and invade, as shown in Figure 6a (at update 2000 direct‐pcd‐3 mean kin unicells without PCD gene is 4.72, direct‐pcd‐100 mean kin unicells without PCD gene is 18.31, pairwise Wilcox test *p*‐value = 8.988e‐11).

When the benefit is instead indirect, programmed cell death is only beneficial if a sufficient number of unicells are considered nonkin, as shown in Figure 5b. A kin inclusivity level of zero harms the maximum number of unicells with a programmed cell death event (mean 14.23 unicells affected per PCD event at update 500). When the kin inclusivity level of an indirect benefit increases to three (indirect‐pcd‐3), the number of unicells harmed significantly decreases (mean 0.27 unicells affected per PCD event at update 500, pairwise Wilcox test *p*‐value = 5.332e‐10), thereby decreasing the benefit of the trait. This effect is even more extreme at indirect‐pcd‐100 where no unicells are considered nonkin at update 500, making the programmed cell death worthless. As shown in Figure 6b, indirect‐pcd‐0 also prevents unicells without the PCD gene from invading throughout evolution.

As Dawkins discussed in The Selfish Gene, whether an altruistic trait is selected for ultimately depends on whether it is able to increase the number of copies of itself in the population (Dawkins, 2006). Relatedness in Hamilton's Rule is a proxy for the likelihood of an organism containing an altruistic gene (Hamilton, 1964), but here we are able to directly measure the effect on unicells containing the altruistic gene of interest. We have shown that the degree of kin discrimination affects kin selection differently depending on whether the programmed cell death behavior directly affects kin or nonkin. When the behavior is indirect, the lowest level of kin inclusivity is beneficial initially for a large effect and later in evolution to resist invasion. However, if the benefit is direct, there is a trade‐off between high initial benefit by having high kin inclusivity and the ability to resist subsequent invasion, which requires a lower kin inclusivity level. Due to the wide variety of kin discrimination mechanisms and direct or indirect benefits found in organic systems (Bidle, 2016; Fiegna & Velicer, 2003; Fukuyo, Sasaki, & Kobayashi, 2012), these varying responses should be taken into consideration when analyzing programmed cell death behavior.

## 5 CONCLUSIONS

We have shown in this work that programmed cell death can evolve due to kin selection under conditions where it otherwise would not have evolved. This work provides a proof of concept that kin selection can in fact be the driving force behind the evolution of programmed cell death in unicellular organisms. We further analyzed the effects of whether the benefit of programmed cell death was direct or indirect and the degree of kin discrimination. We found that accurate kin discrimination was necessary for kin selection to evolve programmed cell death. However, programmed cell death that confers a direct benefit to kin evolved to significantly higher use when many unicells are considered kin whereas when the benefit was indirect, the most extreme kin discrimination led to the highest use of programmed cell death.

While it is difficult to fully control organic systems, several experimental systems have suggested that programmed cell death could have been under direct selection due to inclusive fitness including *E. coli* (Refardt et al., 2013), *Dictyostelium discoideum* (Matapurkar & Watve, 1997), and *Streptococcus mutans* (Perry, Cvitkovitch, & Levesque, 2009). Furthermore, while this work demonstrates that programmed cell death can arise as the result of benefits conferred to kin, it does not rule out other factors and conditions that may result in the evolution of programmed cell death as well. Indeed, further explorations using this system could test the alternative mechanisms that may lead to the evolution of programmed cell death.

The presence or de novo evolution of programmed cell death in unicellular organisms is an exciting possible mechanism for improving human health, either by triggering programmed cell death in pathogenic bacteria or to reduce viral load in beneficial bacteria. However, to harness that power, we must understand what selective forces are acting on the behavior now and in the past. This work contributes to our understanding of how such behavior could have evolved and provided a system that can be used to understand how it will continue to change.

## CONFLICT OF INTEREST

None declared.

## AUTHOR CONTRIBUTIONS

All authors contributed to planning the experiments, implementing the software, analyzing the results, and writing the manuscript. Additionally, Vostinar conducted all experiments.

## Data Availability

Data analysis available at: https://github.com/anyaevostinar/SuicidalAltruismDissertation/tree/master/LongTerm

## ENDNOTE

1 An update is the time unit in Avida during which unicells execute, on average, 30 instructions.
